# Horse gram (*Macrotyloma uniflorum*) as a climate-resilient legume with functional and nutraceutical potential

**DOI:** 10.3389/fpls.2026.1891496

**Published:** 2026-08-07

**Authors:** Amrita Halder, Utpal Das, Jeevitha Gada Chengaiyan

**Affiliations:** 1 Vellore Institute of Technology (VIT) School of Agricultural Innovations and Advanced Learning (VAIAL), Vellore Institute of Technology, Vellore, Tamil Nadu, India; 2 School of Bio Sciences and Technology (SBST), Vellore Institute of Technology (VIT), Vellore, Tamil Nadu, India

**Keywords:** antinutritional factors, bioactive compounds, functional foods, *Macrotyloma uniflorum*, nutraceutical potential

## Abstract

*Macrotyloma uniflorum* (horse gram) is a neglected and climate-resilient leguminous food crop that has great potential to contribute toward global food and nutritional security, sustainability of food systems, and human health. The present review discusses the nutritional, bioactive components, antinutritional factors, processing technologies, techno-functional properties, nutraceutical potential, and climate resilience of *M*. *uniflorum*, with special reference to its novel application as a functional food. Horse gram is rich in protein, fiber, minerals, vitamins, phenolics, flavonoids, anthocyanins, and bioactive peptides, and possesses antioxidant, anti-inflammatory, antidiabetic, cardioprotective, antihypertensive, and antimicrobial properties. Research on customary and novel processing methods has contributed to understanding its bioavailability and the effect of antinutritional factors in processed horse gram products. This review is unique in collating information relating to climate resilience, bioactive peptides, processing technologies, and the likelihood of precision nutrition and nutraceuticals in the future. Despite the abundance of data from *in vitro*, *in vivo*, and preclinical studies supporting the health benefits of horse gram, a limited number of human population-based studies have been published. Gaps in knowledge include bioavailability, mechanisms of action, gene–diet interactions, techno-functional properties, scalability, and industry regulation. Future studies will rely on multi-omics approaches, nutrigenomics, clinical testing, and scalable processing methods to establish horse gram as an evidence-based and commercially useful functional food resource.

## Graphical Abstract

**Figure:**
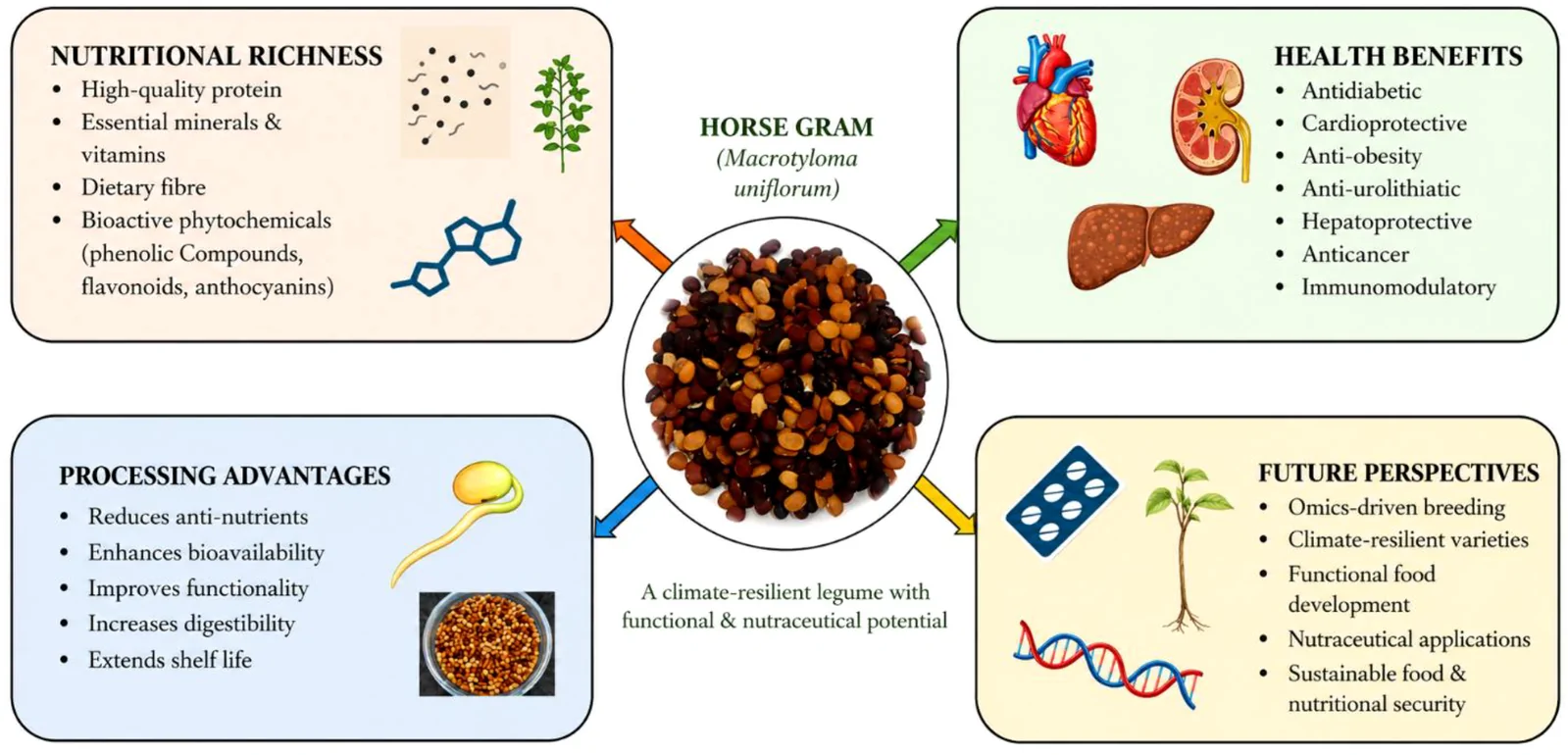
Graphical Abstract of horse gram (*Macrotyloma uniflorum*) as a climate-resilient legume

## 1 Introduction

*Macrotyloma uniflorum*, commonly known as horse gram and previously classified as *Dolichos biflorus* L., is an unexplored leguminous crop with many beneficial properties. It is distributed mainly in Southeast Asia and tropical Africa and is cultivated during the late rainy season (August to October). The name “Macrotyloma” originates from the Greek words “makros”, meaning large, “tylos”, meaning knob, and “loma”, meaning margin, which refers to the knobby structures on its pods. It is commonly called “hulga”, and is used for both food and fodder (Ingle et al., 2020; Kashid et al., 2021). It is locally known as kulthi, muthira, hurali, madras gram, and gaheth. It has been classified as a “potential food source of the future” by the U.S. National Academy of Sciences due to its resilience, drought tolerance, and nutritional value (Chirania and Sharma, 2021). It is a hardy pulse crop of the semi-arid tropics that has been poorly studied. It is one of the more resilient pulse crops within the semi-arid tropics. Compared with popular legume crops such as chickpeas, mung beans, and pigeon peas, it has a higher tolerance to drought, fluctuations in soil and air moisture, and low soil fertility and moisture conditions. Although these traits are favorable, its high resilience has contributed to its status as an underutilized and poorly researched pulse crop (Aditya et al., 2019). Despite its current and historical importance in the diets of a large proportion of the population in India, entrenched biases exist against horse gram because it is considered a low-status food of the poor, particularly in southern India (Fuller and Murphy, 2018). Hence, it is often referred to as the “poor man’s meat”, especially in developing countries (Suwarna et al., 2019). It is well adapted to less fertile and stressful environments and is cultivated around the world, mainly in India, Burma, Sri Lanka, Australia, Nepal, and Africa. In India, it is grown mainly in Himachal Pradesh, Sikkim, Maharashtra, Andhra Pradesh, Karnataka, and Tamil Nadu (Gautam et al., 2020a). In addition, significant cultivation is reported from Odisha, Chhattisgarh, West Bengal, Bihar, Himachal Pradesh, and Uttarakhand, reflecting its adaptability to both dryland and hilly terrains. Its resilience to marginal environments makes it an important pulse crop in regions where other legumes fail to thrive (Jayashree and Krishnamma, 2020). Horse gram is a significant climate-resilient grain legume grown throughout the varied agro-climatic conditions in India, especially in the dry tropics. It is a low-input crop compared with other pulses. Typically grown under moderate rainfall conditions, horse gram exhibits strong resistance to pests and diseases, contributing to its suitability for marginal farming systems. **Table 1** summarizes the climate resilience traits of horse gram. India dominates global horse gram cultivation, with production estimated at 2.62 lakh tonnes on approximately 5.07 lakh hectares. Karnataka, Andhra Pradesh, Tamil Nadu, Odisha, Maharashtra, and Chhattisgarh are India’s major horse gram-growing regions. Horse gram thrives in most resource-limited and semi-arid production systems. Not only does horse gram supply subsistence and functional food needs, but it is also emerging in markets. India’s horse gram exports include Sri Lanka (58.03%), the United Arab Emirates (10.22%), and Canada (10.17%), demonstrating increasing demand for this legume and its value-added products (Indian Institute of Pulses Research, 2024). It is consumed as whole seeds or used as raw material for food processing. It represents an underutilized legume with great potential for human nutrition, particularly for rural communities, and is cultivated in temperate and subtropical regions (Linn et al., 2018). It has been recognized as a potential source of protein, different essential amino acids, carbohydrate, soluble and insoluble dietary fiber, minerals such as Fe, Mg, Cu, Mo, Ca, and Zn, and vitamins such as thiamine, niacin, and riboflavin, making it highly suitable for functional and nutraceutical applications (Malik et al., 2025; Suwarna et al., 2019; Vashishth et al., 2018). It contains different bioactive compounds, including flavonoids (quercetin, myricetin, and kaempferol), anthocyanins (cyanidin and malvidin), and phenolic compounds (gallic acid, syringic acid, and p-coumaric acid), which exhibit various important physiological beneficial activities such as radical scavenging, antimutagenic, anticholesteromic, and anticarcinogenic effects (Oli et al., 2024). Although horse gram is recognized as an important nutritional and agronomic crop, it has not received the same level of research as major grain legumes. Research gaps include limited information on bioavailability, molecular mechanisms of action, clinical efficacy, and industrial applications. This review critically summarizes current knowledge on nutritional, functional, and nutraceutical properties of horse gram, along with potential future prospects for developing functional foods and sustainable nutrition strategies. Advanced processing technologies, multi-omics technologies, and nutrigenomics are proposed to further enhance its value as a climate-resilient functional food crop.

**Table 1:** Climate resilience traits of horse gram.

Trait	Horse gram response	Agricultural significance	References
Drought tolerance	Hardy, drought-tolerant legume with identified QTLs for drought-related traits	Enables cultivation in water-scarce regions; multiple physiological mechanisms have been identified	(Bhardwaj et al., 2013; Katoch and Chahota, 2021; Priyanka et al., 2019)
Low water requirement	Grows under rainfed situations	Reduces irrigation dependency and is suitable for climate-vulnerable regions	(Sahoo and Mohanty, 2020)
Pest resistance	Exhibits natural defense mechanisms through protease inhibitors and possesses identified resistance/tolerance sources against powdery mildew, anthracnose, and yellow mosaic disease	Reduces crop losses and pesticide dependence while supporting resilient cultivation under low-input conditions	(Mahesh et al., 2021; Rajaprakasam et al., 2023)
Marginal soil adaptability	Successfully cultivated in marginal lands	Expands cultivation to resource-poor regions and improves soil fertility through nitrogen fixation	(Priyanka et al., 2019; Sahoo and Mohanty, 2020)

## 2 Nutritional composition

Several studies have reported that *M. uniflorum* exhibits superior nutritional characteristics compared with other popular food pulses, including high protein content, higher mineral density, and higher concentrations of bioactive compounds. These findings have been reported consistently across multiple independent studies, strengthening the evidence for its excellent nutritional profile. This holistic nutritional profile positions *M. uniflorum* as a rich source of nutrients and a valuable crop for combating malnutrition and for developing functional foods with nutritional and therapeutic applications, supported by substantial scientific evidence from numerous analytical studies (Rana et al., 2023).

**Figure 1:**
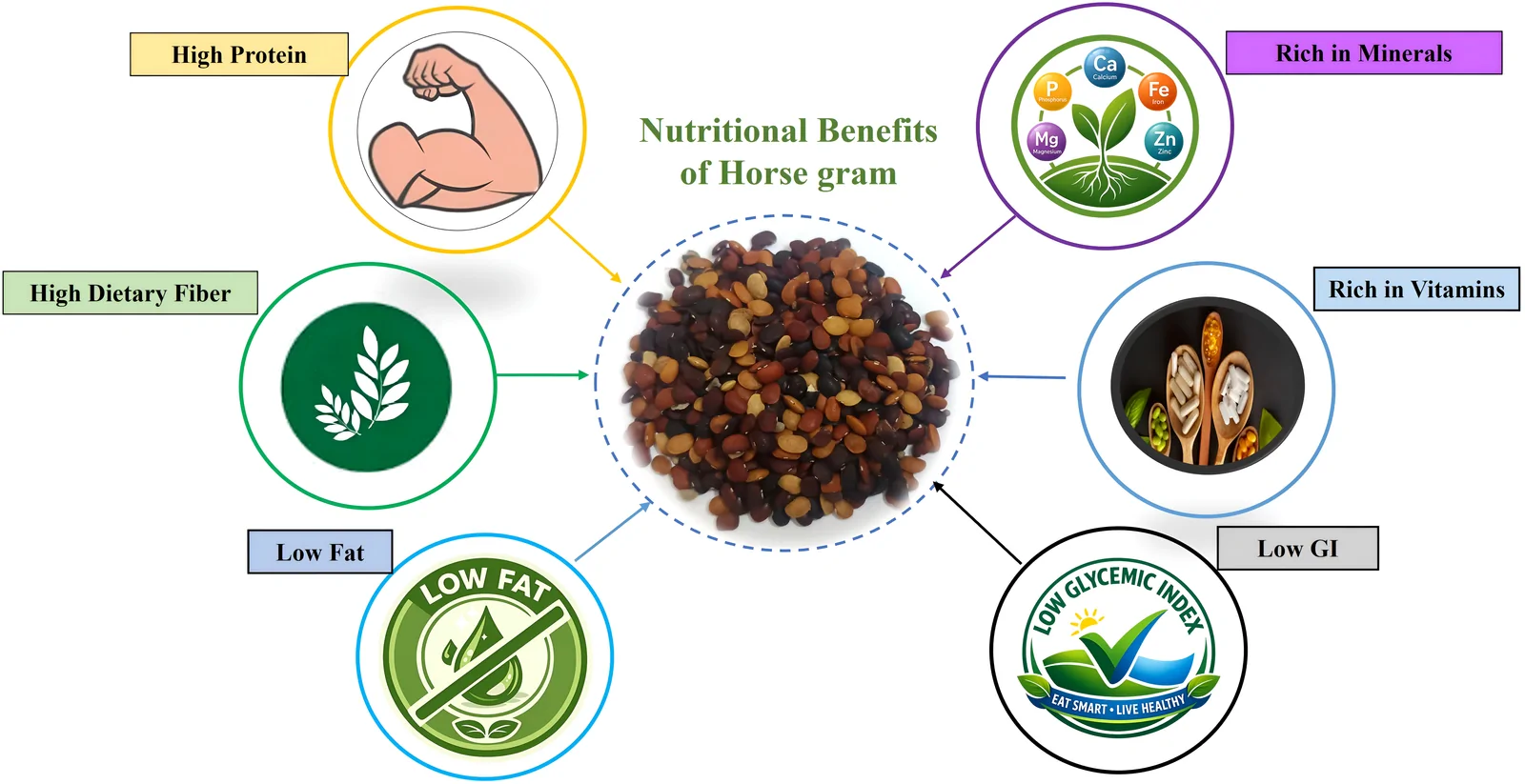
Nutritional composition of horse gram seeds.

The introduction of *M. uniflorum* into modern food systems is not only an opportunity from an agricultural perspective but also represents a paradigm shift toward achieving sustainable food and nutrition security while incorporating traditional knowledge and addressing contemporary challenges. Horse gram exhibits a distinctive nutritional profile compared with other commonly consumed pulses (**Table 2**). It is rich in protein (21.73 g/100 g), which is similar to that of green gram and slightly lower than that of lentil, making it an important source of plant-based protein for growth, tissue repair, and metabolic activities. It is a low-fat crop (0.62 g/100 g) that could benefit cardiovascular health and be incorporated into low-calorie diets. Horse Gram is also an excellent source of carbohydrates (57.24 g/100 g), which provide a slow-release source of energy. It contains dietary fiber, which can assist in digestion, help regulate blood sugar levels, and help decrease cholesterol levels. It is a legume particularly rich in essential minerals. For example, 12.14 mg of horse gram would provide 100% of the Recommended Dietary Allowance for iron, while 269 mg would supply the daily requirement for calcium, and 298 mg would supply the daily requirement for phosphorus. Additionally, 100 g of horse gram contains 1,065 mg of potassium. It also contains B-complex vitamins, including thiamine, riboflavin, and niacin. Overall, horse gram is a nutrient-rich legume with considerable potential as both a functional food and a nutraceutical. It can also be used in food security programs (Longvah et al., 2017). *Macrotyloma uniflorum* (Lam.) Verdc., an underutilized legume, is attracting renewed scientific interest because of its potential to contribute to sustainable solutions for global food security (Sah et al., 2024). Recent research has provided substantial new insights into the nutritional value and bioactive components of this drought-tolerant legume, highlighting its potential to help multiple nutritional gaps (Gautam et al., 2020b; Kalpanadevi and Mohan, 2013; Nagarjun et al., 2025). **Figure 1** shows the nutritional composition of horse gram.

**Table 2:** Comparative nutritional profile of horse gram and major pulses.

Composition	Horse gram (*Macrotyloma uniflorum*)	Chickpea/bengal gram (*Cicer arietinum*)	Red/pink lentil (*Lens culinaris*)	Cowpea (*Vigna unguiculata*)	Green gram (*Vigna radiata*)
Energy (kcal)	329 ± 9	287 ± 10	322 ± 11	320 ± 7	294 ± 10
Protein (g/100 g)	21.73 ± 0.29	18.77 ± 0.42	24.35 ± 1.10	20.36 ± 0.59	22.53 ± 0.43
Fat (g/100 g)	0.62 ± 0.04	5.11 ± 0.11	0.75 ± 0.04	1.15 ± 0.06	1.14 ± 0.10
Carbohydrates (g/100 g)	57.24 ± 0.50	39.56 ± 0.16	52.53 ± 1.05	54.62 ± 0.49	46.13 ± 0.64
Dietary fiber (g/100 g)	7.88 ± 0.02	25.22 ± 0.39	10.43 ± 0.39	11.54 ± 0.13	17.04 ± 0.38
Moisture (g/100 g)	9.28 ± 0.57	8.56 ± 0.37	9.71 ± 0.48	9.42 ± 0.39	9.95 ± 0.42
Ash (g/100 g)	3.24 ± 0.11	2.78 ± 0.13	2.23 ± 0.13	2.90 ± 0.11	3.22 ± 0.04
Calcium (mg/100 g)	269 ± 34.9	267 ± 21.9	310 ± 5.3	372 ± 32.9	353 ± 33.6
Iron (mg/100 g)	12.14 ± 0.17	6.26 ± 0.60	10.27 ± 0.07	13.68 ± 0.07	12.48 ± 0.07
Phosphorus (mg/100 g)	298 ± 22.5	267 ± 21.9	310 ± 5.3	372 ± 32.9	353 ± 33.6
Magnesium (mg/100 g)	152 ± 18.1	160 ± 17.5	74.69 ± 7.78	213 ± 20.3	198 ± 39.2
Potassium (mg/100 g)	1065 ± 42.4	935 ± 37.9	786 ± 55.3	1241 ± 116	1177 ± 74.3
Zinc (mg/100 g)	2.71 ± 0.21	3.37 ± 0.26	3.61 ± 0.26	3.41 ± 0.41	2.67 ± 0.13
Niacin (mg/100 g)	1.82 ± 0.26	2.10 ± 0.06	1.81 ± 0.02	1.64 ± 0.03	2.11 ± 0.13
Thiamine (B1) (mg/100 g)	0.32 ± 0.02	0.37 ± 0.04	0.34 ± 0.03	0.33 ± 0.07	0.45 ± 0.27
Riboflavin (B2) (mg/100 g)	0.24 ± 0.03	0.24 ± 0.11	0.16 ± 0.05	0.09 ± 0.09	0.27 ± 0.11
Vitamin B6 (mg/100 g)	0.21 ± 0.017	0.36 ± 0.025	0.18 ± 0.022	0.32 ± 0.033	0.35 ± 0.034
Folate (B9) (µg/100 g)	163 ± 5.3	233 ± 12.9	49.99 ± 4.91	231 ± 27.3	145 ± 5.4
Tocopherols	0.06 ± 0.01	1.59 ± 0.07	0.03 ± 0.00	0.33 ± 0.02	0.13 ± 0.02

Source: (Abebe and Alemayehu, 2022; Arya and Divya, 2025; Baptista et al., 2017; Begum et al., 2023; Dhull et al., 2023; Longvah et al., 2017).

### 2.1 Protein

*Macrotyloma uniflorum* demonstrates a high protein content, with multiple independent studies reporting consistent values ranging from 15%–22%. A study by Kalpanadevi and Mohan (2013) documented a crude protein content of 21.50% in seeds collected from Eastern Ghats, Tamil Nadu, while another study by Marimuthu and Krishnamoorthi (2013) reported a crude protein content of 22.12%. Jain et al. (2012) found protein contents of 15.10% and 15.32% in the AK-21 and AK-42 varieties, respectively. This protein content significantly exceeds that of many commonly consumed cereals and compares favorably with other legumes (Bhartiya et al., 2015).

**Figure 2:**
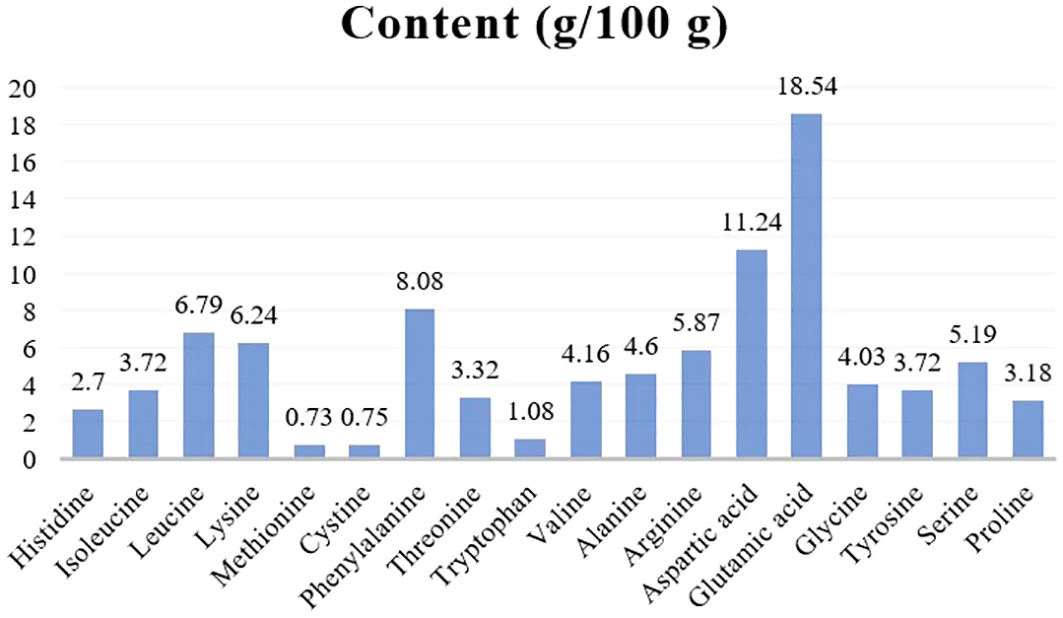
Amino acid content in horse gram seeds (Longvah et al., 2017).

It contains appreciable levels of essential amino acids, such as lysine, leucine, valine, histidine, and arginine, contributing to its nutritional value as a plant-based protein source (Arya and Divya, 2025). **Figure 2** shows the amino acid content in horse gram seeds.

Horse gram proteins and peptides are considered to promote healthy body composition and help maintain healthy blood sugar levels. These proteins also appear to exhibit antioxidant and anticancer activities, support a healthy immune system, and promote cardiovascular health (Oli et al., 2024).

Horse gram seeds contain a heterogeneous protein complement dominated by albumin and globulin fractions. Biochemical and electrophoretic studies have identified multiple storage protein classes, including vicilin, legumin, narbonin, and Bowman–Birk type proteinase inhibitors, together with several polypeptide bands spanning approximately 17–186 kDa. These findings indicate that horse gram seed protein is distributed across multiple subunits rather than a single purified storage-protein species (Gautam and Chahota, 2024; Sharma et al., 2022).

Through protein characterization studies, many protease inhibitor subunits of the Bowman–Birk inhibitor family have been discovered. Analysis shows that some of these inhibitors exist as monomers (HGGI-I, HGGI-II, and HGGI-III) and dimers (HGI-III). Research on stimuli response has shown that the presence of dimers (intermolecular) is due to specific interactions of amino acids, providing valuable insight into the diversity of protein structures and their functions (Sreerama and Gowda, 1998).

### 2.2 Fat

The lipid content of horse gram ranges from 0.6%–2.6%, according to recent analyses. Whole horse gram seeds contain 0.70%–2.06% crude fat, while dehulled seeds show higher content (0.81%–2.11%) (Rana et al., 2023). The fatty acid profile demonstrates nutritional superiority with 72.49% unsaturated fatty acids, comprising 42.78% linoleic acid, 16.15% oleic acid, and 13.56% linolenic acid. Saturated fatty acids constitute 27.5%, including 21.97% palmitic acid, 2.85% arachidic acid, 2.32% stearic acid, and 0.36% myristic acid (Sah et al., 2024).

Despite its low lipid content, horse gram is a valuable source of essential unsaturated fatty acids. These fatty acids have a positive impact on health, including an indirect reduction of protein glycosylation, alleviating oxidative stress, and exerting antiulcer, cardioprotective, and anti-inflammatory effects. All of these enhance the nutraceutical value of horse gram (Mishra et al., 2012; Mishra and Pathan, 2011).

### 2.3 Carbohydrates

Carbohydrate content varies between 51.9%–60.9% for whole seeds and 56.8%–66.4% for dehulled seeds. Raw horse gram seeds contain 36 ± 1.17 g starch per 100 g of dry matter, with approximately 85% digestible starch, 14.47% resistant starch, and 3.38% resistant starch associated with insoluble dietary fibers. Recent comprehensive analyses have revealed high total dietary fiber fibercontent (28.8%) fiber, consisting of 27.82% insoluble dietary fiber (IDF) and 1.13% soluble dietary fiber (SDF), with an IDF to SDF ratio of 24.6 (Rana et al., 2023; Sah et al., 2024).

The properties of the dietary fibers in horse gram enhance digestive health, help reduce cholesterol levels, and may lower the risk of cardiovascular and gastrointestinal diseases. Its high fiber content also contributes to its potential functional food and anticancer properties (Sreerama et al., 2010).

Carbohydrates in horse gram contribute to glycemic control and gastrointestinal health. They may also enhance the availability of phytochemicals and micronutrients and have been associated with anti-inflammatory, anti-coagulant, and angiogenesis-regulating activities, further supporting the functional food potential of horse gram (Bhokre et al., 2015; Morris et al., 2013).

### 2.4 Moisture and ash content

Whole horse gram seeds contain 11.55% moisture and 3.0%–3.8% ash content, while dehulled seeds contain 9.73% moisture and 2.7%–3.4% ash content (Bhartiya et al., 2015; Sah et al., 2024).

### 2.5 Mineral and vitamin composition

The mineral composition of horse gram (*Macrotyloma uniflorum*) demonstrates its significance as a nutrient-dense legume, particularly in terms its macro- and micromineral content. Recent profiling studies indicate that macrominerals are present in the range of 1.3–14 mg/g dry weight, with calcium being one of the predominant elements, reported at 238–312 mg/100 g in whole seeds and slightly lower concentrations in dehulled samples. Iron content ranges between 5.89–7.44 mg/100 g, with *in vitro* bioavailability studies suggesting available fractions of 22.50–38.50 mg calcium and 0.26–0.85 mg iron per 100 g. Phosphorus is also present at appreciable levels (311 mg/100 g), along with significant concentrations of potassium, magnesium, and sulfur, which collectively contribute to metabolic and physiological functions. These minerals play vital roles in bone and tooth development, blood clotting, muscle contraction, and the transmission of neurotransmitters. They also play a role in maintaining immune system health and helping in antiviral and antibacterial defense systems (Carter et al., 2021; Morris et al., 2013). Moreover, horse gram also contains microminerals, including zinc, copper, manganese, and nickel, etc., which support enzymatic activity and antioxidant defense systems. These minerals are present at concentrations ranging from 1.0 to 95.0 μg/g dry weight (Bhartiya et al., 2015; Morris et al., 2013). Moreover, it is an abundant source of B-complex vitamins, including thiamine, riboflavin, niacin, vitamin B6, and folic acid, further enhancing its nutritional value (Longvah et al., 2017). The vitamin profile indicates that the vitamin B1 (Thiamine) content was 0.38 mg/100g, B2 (riboflavin) content was 0.19 mg/100g, and B3 (niacin) content was 1.42 mg/100g. These B-complex vitamins help support cell proliferation, immune system function, and cholesterol metabolism. They also help in antioxidant activities, prevent anemia by red blood cell formation, and promote heart health (Amir Maqbool et al., 2017; RLDS, 2017). All these findings indicate that horse gram has a considerable potential as an important source of minerals and vitamins for the development of functional and therapeutic foods (Ingle et al., 2020).

## 3 Bioactive compounds

Horse gram is a good source of various bioactive compounds (flavonoids, phenolic acids, tannins, phytosterols, and saponins). These bioactive molecules play a crucial role in the antioxidant, anti-inflammatory, antihyperglycemic, and hypolipidemic activities of the legume. Horse gram is known for its nutritional richness and its wide range of bioactive compounds, which offer multiple health benefits and nutraceutical potential. These compounds exhibit a wide range of pharmacological properties, making horse gram a valuable functional food and traditional remedy. The key bioactive compounds and pharmacological functions present in horse gram are shown in **Table 3**.

**Table 3:** Bioactive compounds and their molecular pharmacological functions.

Class	Bioactive compound	Approx. quantity (µg/g DW)*	Key pharmacological benefit (one-line)	Chemical structure	References
Phenolic acids	Gallic acid	5.5–26.9	Potent antioxidant with anticancer and cardioprotective effects	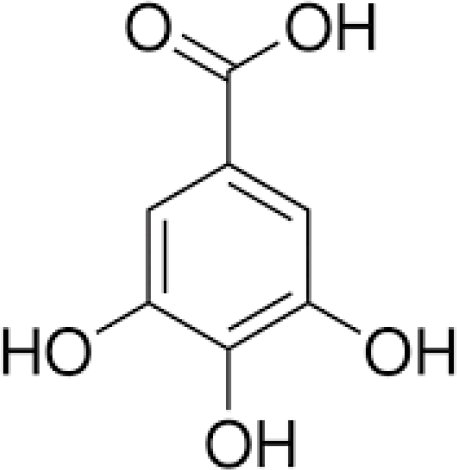	(Bai et al., 2021; Bhuia et al., 2023; Xu et al., 2021)
	Syringic acid	4.5–18.4	Neuroprotective and anti-inflammatory compound with hepatoprotective action	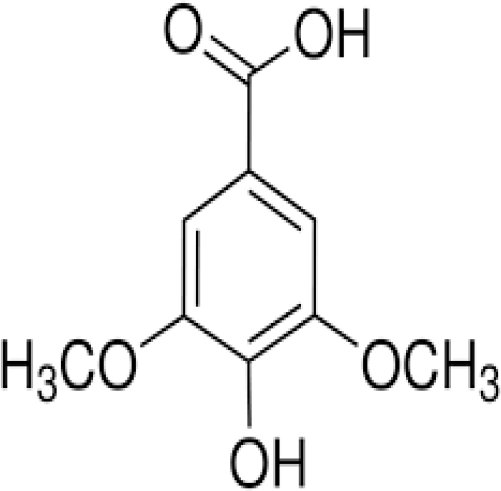	(Ogut et al., 2022; Srinivasulu et al., 2018)
	Caffeic acid	10.6–88.9	Strong antioxidant with antimicrobial and anti-aging potential	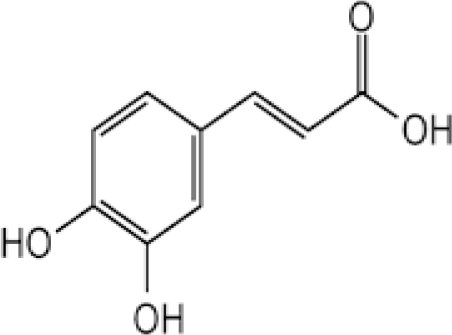	(Alam et al., 2022; Magnani et al., 2013)
	Sinapic acid	3.7–9.7	Provides antioxidant and anti-inflammatory activity with neuroprotection	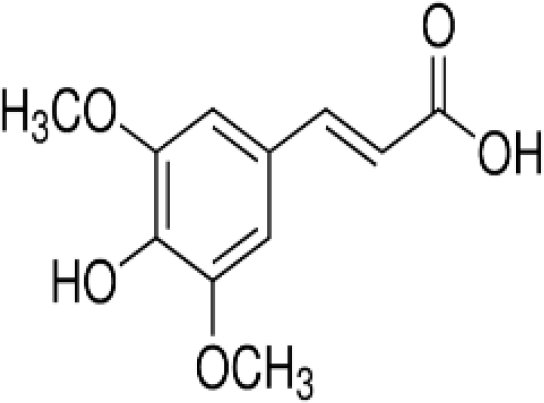	(Chu et al., 2021)
	Vanillic acid	42.4–58.4	Shows neuroprotective, antidepressant, and antioxidant effects	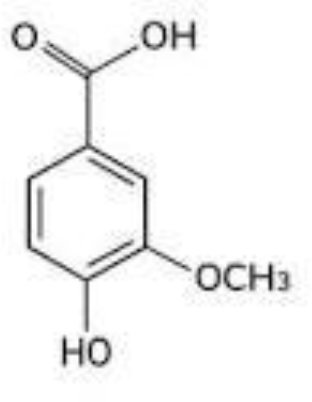	(Ingole et al., 2021)
	p-Coumaric acid	21.4–40.9	Antidiabetic and antihyperlipidemic activity, aiding metabolic health	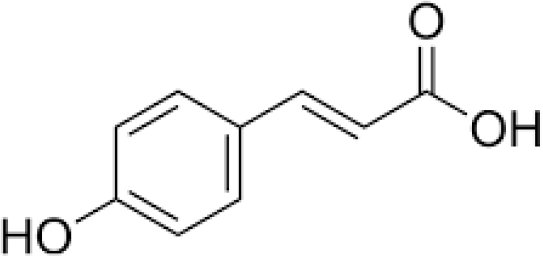	(Boz, 2015)
	p-Hydroxybenzoic acid	13.1–28.8	Exhibits antimicrobial, antiviral, and anti-inflammatory properties	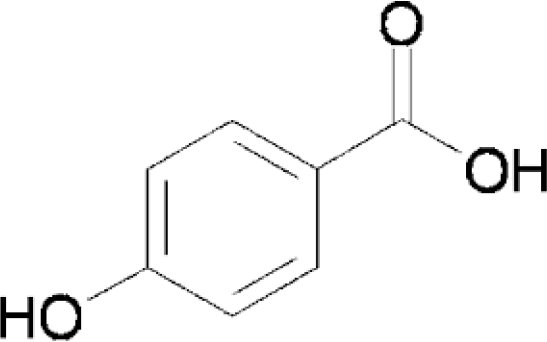	(Xu et al., 2021)
	Ferulic acid	31.4–70.1	Known for antioxidant, anticancer, and hepatoprotective effects	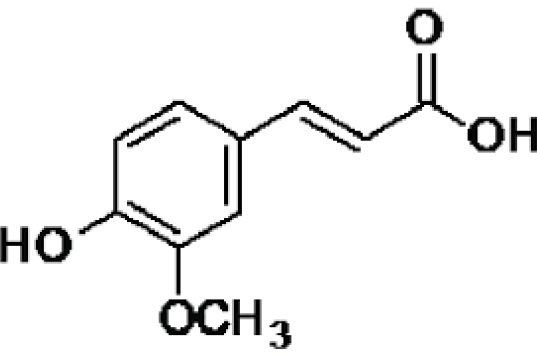	(Rukkumani et al., 2004)
	Protocatechuic acid	11.8–39.0	Provides antihyperglycemic and anti-inflammatory activity	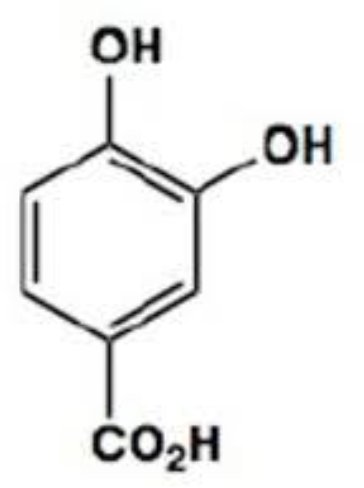	(Semaming et al., 2015)
Flavonoids	Quercetin	9.7–129.5	Strong antioxidant with cardioprotective and antiobesity effects	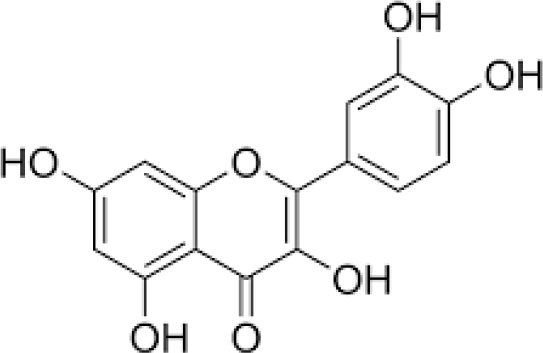	(Nabavi et al., 2015)
	Kaempferol	6.0–117.2	Anti-inflammatory, anticancer, and hepatoprotective flavonoid	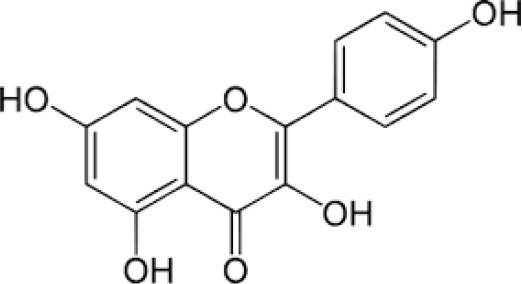	(Imran et al., 2019)
	Myricetin	2.4–35.5	Antidiabetic and neuroprotective compound regulating glucose metabolism	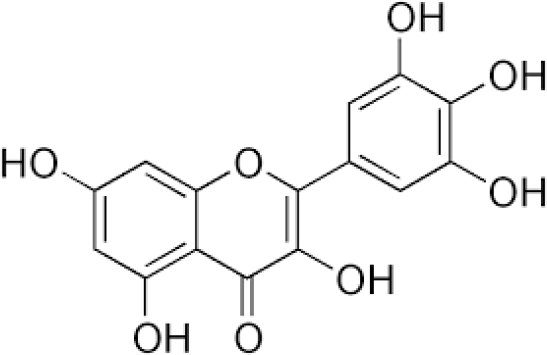	(Song et al., 2021)
	Daidzein	0.94–22.2	Phytoestrogen with cardioprotective and antiosteoporotic effects	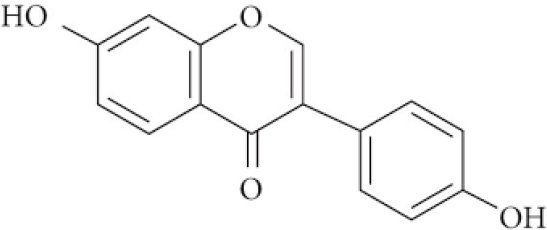	(Alshehri et al., 2021; Sun et al., 2016)
	Genistein	44.7 (major in embryo)	Anticancer compound with hormone-modulating activity	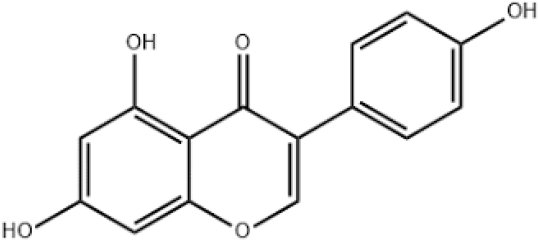	(Weng et al., 2019)
Anthocyanins	Cyanidin	41.6–175.8	Antioxidant and anti-inflammatory pigment with a cardioprotective role	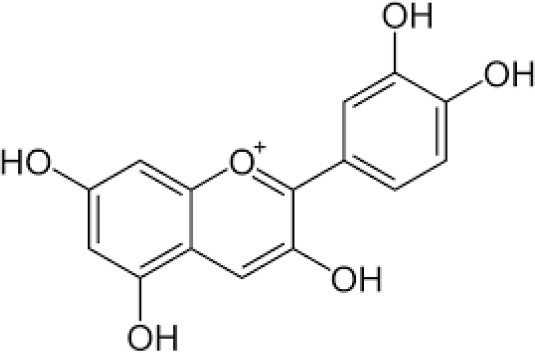	(Deepa et al., 2023)
	Delphinidin	21.4–408.7	Cell-protective compound with anti-angiogenic and anticancer potential	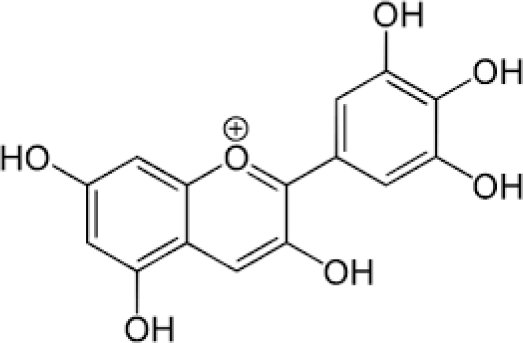	(Oli et al., 2024)
	Malvidin	9.7	Exhibits anti-inflammatory and cardioprotective properties	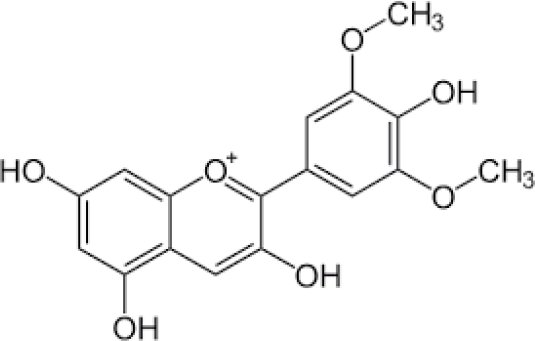	(Oli et al., 2024)

The use of horse gram as an important source of bioactive peptides is of recent origin. The application of the Alcalase enzyme to horse gram resulted in the isolation of two bioactive peptides, TVGMTAKF and QLLLQQ, that were found to maintain their bioactivity through simulated gastrointestinal digestion and could therefore be considered useful for antihypertensive purposes (Bhaskar et al., 2019).

Enzymatic hydrolysis of horse gram proteins using enzymes enhances protein solubility and gives rise to hydrolysates with antioxidant and ACE-inhibiting properties. Peptides obtained from these proteins have good digestibility and techno-functional properties (Oli et al., 2024; Sharma and Thakur, 2025).

### 3.1 Phenolic acids and flavonoids

Horse gram contains various phenolic acids such as gallic acid, syringic acid, caffeic acid, chlorogenic acid, sinapic acid, vanillic acid, p-coumaric acid, p-hydroxybenzoic acid, and protocatechuic acid. Flavonoids such as quercetin, daidzein, myricetin, kaempferol, and genistein are also present. These compounds are crucial for horse gram’s antioxidant, antidiabetic, anti-inflammatory, and anticancer effects (Oli et al., 2024; Sreerama et al., 2010).

### 3.2 Anthocyanins

The anthocyanins present in horse gram seeds include cyanidin, petunidin, malvidin, and delphinidin, which amongcontribute to horse gram’s antioxidant and anti-inflammatory properties (Oli et al., 2024; Sreerama et al., 2010).

### 3.3 Fatty acids

Fatty acids such as linoleic acid and linolenic acid are present, which are important for treating cardiovascular and diabetic conditions and for inhibiting Parkinson’s and Alzheimer’s diseases. Stearic acid, arachidic acid, palmitic acid, and oleic acid are also found (Oli et al., 2024; Sreerama et al., 2010).

### 3.4 Other phytochemicals

The seed also contains tannins, urease, glycosides, beta-sitosterol, sterols, phytic acid, and saponins. LC-MS analysis has revealed compounds such as syringic acid hexoxide (antidiabetic), tyramine (blood regulation), proline, betaxanthin (body temperature regulation), and rutaecarpine (cardiac anaphylactic injury). GC-MS analysis of the seed extract showed 38 phytochemicals, including pentadecanoic acid, hexadecenoic acid, and heptadecanoic acid, known for their antioxidant, nematicidal, pesticidal, lubricant, and hypocholesterolemic properties (Oli et al., 2024).

## 4 Antinutritional factors and their effects on digestibility

*Macrotyloma uniflorum* exhibits the presence of several antinutritional compounds that adversely affect nutrient bioavailability and digestive enzyme activity. Quantitative analysis reveals significant concentrations of antinutritional factors in unprocessed horse gram flour, with tannin content measured at 7.9 mg/g and phytate content at 0.96 mg/g (Sarvani et al., 2020). The predominant antinutritional constituents identified include tannins, phytic acid, oxalates, polyphenolic compounds, trypsin inhibitors, lectins, and protease inhibitors. These bioactive compounds are found to inhibit nutrient uptake and affect the digestive process. Specifically, trypsin inhibitors significantly reduce protein digestibility (Pagar et al., 2021; Haripriya et al., 2017).

Studies on the distribution of antinutritional factors in horse gram have shown that these compounds are mainly deposited in the seed coat fractions, while phytic acid is mainly deposited in the cotyledon fractions. Phenolic compounds in the horse gram seed coat exhibited inhibitory effects on digestive enzymes. Kinetic studies confirm that the phenolics present in the seed coat inhibit α-amylase in mixed (non-competitive) inhibition mode, whereas α-trypsin is inhibited both in mixed (non-competitive) and uncompetitive manner (Sreerama et al., 2010). Such enzymatic inhibition directly affects the digestibility of carbohydrates and proteins. Genetic variability analysis among the mutants clearly showed a high coefficient of variation, total phenolic content (17.24%), tannins (10.88%), phytic acid (16.14%), and oxalic acid (15.16%) (Sudhagar et al., 2023).

Compared with other commonly consumed legumes, horse gram exhibits a distinctive antinutritional profile. Sreerama et al. (2012) stated that horse gram has lower levels of phytic acid (10.2 mg/g) compared to chickpea (12.1 mg/g) and cowpea (14.0 mg/g), which may indicate lower mineral-chelating activity. Moreover, the lower IC_50_ value of horse gram (29.4 µg/mL) compared to cowpea (38.2 µg/mL) and chickpea (44.8 µg/mL) may indicate a higher protease inhibitory capacity in horse gram. These inhibitors lower protein digestibility due to their formation of stable complexes with digestive enzymes but, at the same time, are known to provide certain health benefits due to their antioxidant and anticarcinogenic properties. On the other hand, horse gram has lower levels of flatulence-causing oligosaccharides (26.8 mg/g) compared to cowpea (31.7 mg/g) and chickpea (34.9 mg/g). Thus, although horse gram has higher levels of some anti-nutrients, such as polyphenols and trypsin inhibitors, it has lower levels of phytates and oligosaccharides.

**Table 4** shows antinutritional factors and mitigation strategies.

**Table 4:** Antinutritional factors and mitigation strategies.

Antinutritional factor	Adverse effect	Location in seed	Reduction method	Reduction efficiency	References
Phytic acid	Reduces mineral absorption (Ca, Fe, Mg, Zn, Cu, and K); inhibits protein digestibility.	Concentrated in the seed coat and pericarp of grains.	Soaking; pressure cooking; germination; fermentation	12%–14% (pressure cooking); 42%–48% (soaking).	(Mittal et al., 2012; Samtiya et al., 2020)
Tannins	Reduces protein and carbohydrate digestibility; damages digestive mucosa.	Mainly concentrated in the seed coat.	Pressure cooking; boiling; germination; dehulling	93.97% (pressure cooking); 93% (boiling/germination)	(Mittal et al., 2012; Narale et al., 2024; Samtiya et al., 2020)
Oxalic acid	Forms insoluble complexes with Ca and Mg; promotes kidney stone formation; dose lethal at 1,500 mg	Concentrated in the outer layers; highest in leaves and stems.	Boiling (discard water); pressure cooking; blanching	71.79% (pressure cooking); 22%–23% (blanching)	(Fekadu Gemede, 2014; Mittal et al., 2012)
Trypsin inhibitors	Inhibit trypsin and chymotrypsin enzymes; reduce protein digestion and absorption.	Cotyledon (>90% in soy/faba); distributed in cotyledon, embryonic axis, seed coat in chickpea.	Pressure cooking; boiling; autoclaving; roasting.	50% (pressure cooking); 81%–100% (soaking + cooking); 95% (roasting at 180 °C).	(Avilés-Gaxiola et al., 2018; Mittal et al., 2012; Samtiya et al., 2020)

## 5 Impact of processing on nutritional and antinutritional factors

**Figure 3:**
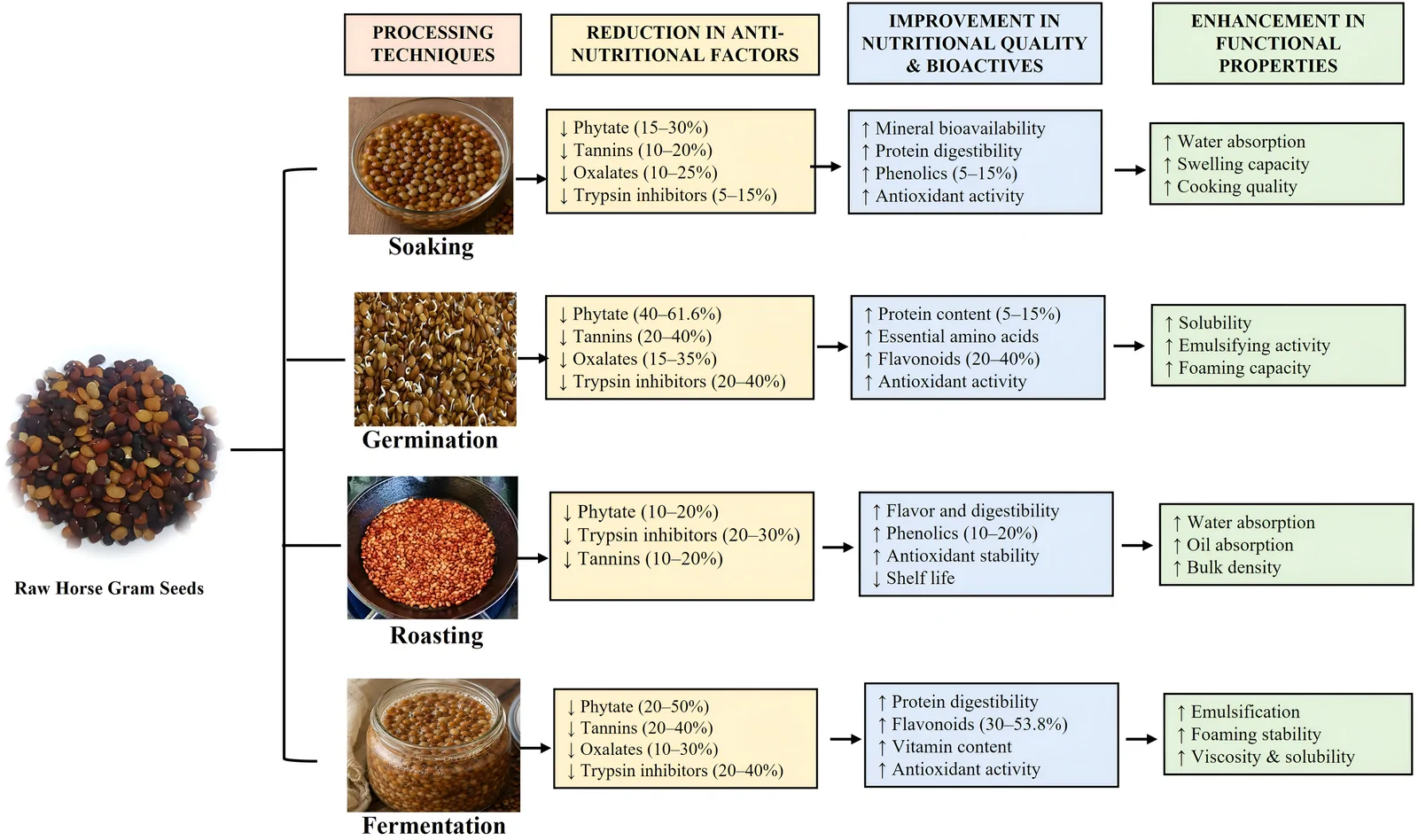
Effects of processing on nutritional and anti-nutritional factors in horse gram seeds.

The nutritional quality and anti-nutrient content of horse gram are significantly affected by processing techniques. Hydration and roasting help break down antinutritional factors such as tannins, oxalates, trypsin inhibitors, and phytates, increasing protein digestibility and mineral bioavailability. Germination is especially effective, with an increase in the levels of phenolics, flavonoids, and antioxidants, and a significant decrease in antinutritional factors due to the enzymatic processes that occur during germination. Fermentation further releases bound phytochemicals, adds microbial metabolites, and enhances antioxidant activity. Advanced technologies such as infrared micronization, microwave processing, and extrusion also help to reduce the antinutritional factors present in horse gram. These processing treatments, when combined with horse gram, result in a more nutritious and bioavailable ingredient that can be used in healthier food formulations. Since conventional processing techniques such as soaking, germination, roasting, and fermentation are inexpensive and simple, they are well suited for use in developing countries because they require few resources. However, advanced processes may not be practical in some countries because they require special equipment and technical knowledge. Therefore, more studies need to be conducted on the scalability, economic feasibility, and technology transfer of these advanced processing techniques in horse gram-producing regions. **Figure 3**, **Table 5** show the effect of processing on nutritional and antinutritional factors.

**Table 5:** Effects of conventional processing on nutritional and antinutritional factors.

Processing method	Protein/nutritional changes	Total phenolics/flavonoids	Tannin reduction	Phytate reduction	Other antinutritional changes	Antioxidant activity	References
Soaking	Increased by 20.66%	Phenolics increased by 28.49%	Reduced significantly	Reduced significantly	Trypsin inhibitor partially reduced	Antioxidant activity decreased by 28.7%	(Ojha et al., 2020; Ul Eain Hyder Rizvi et al., 2022)
Germination	Protein increased by 23.01%	Polyphenols increased by 26.0%; flavonoids increased by 30.7%	Tannins reduced by 54.6%	Phytate reduced by 61.6%	Oxalic acid reduced by 61.6%; trypsin inhibitor reduced by 26.79%	Antioxidant activity increased by 31.51%–51.6%	(Ojha et al., 2020; Pal et al., 2016; Ul Eain Hyder Rizvi et al., 2022)
Fermentation	Improved nutritional quality and flour functionality	Polyphenols increased by 86.9%; flavonoids increased by 53.8%	Tannins reduced by 69.3%	Phytate reduced by 69.5%	Oxalate reduced by 66.7%	Antioxidant activity increased by 59.9%	(Ojha et al., 2020)

### 5.1 Conventional technologies

#### 5.1.1 Soaking

The most basic processing technique for horse gram is soaking. It has been found that horse gram flour is affected to a great extent by soaking in water (1:3 w/v) for up to 24 h, as exhibited by changes in its functional attributes. Soaking, however, also has limitations because it results in a loss of beneficial polyphenols and flavonoids, along with a 28.7% reduction in antioxidant activity (Ojha et al., 2020). Based on optimization studies, a soaking time of 18 h is considered the optimum soaking time for subsequent processing operations (Handa et al., 2017).

#### 5.1.2 Germination

It has been observed that the physical processing method, germination, results in the most effective enhancement of horse gram. Here, soaked seeds are spread on a muslin cloth for 72 h under conditions of 27 °C± 3 °C and 90% relative humidity (RH). Substantial reductions in antinutritional factors upon germination include 61.6% for phytic acid, 54.6% for tannins, and 61.6% for oxalates. At the same time, beneficial phytochemicals increase by 26%, flavonoids by 30.7%, and antioxidant activity by 51.6% (Ojha et al., 2020). Germination is highly dependent on illumination conditions; light exposure is a better alternative for increasing the nutritional benefits (Handa et al., 2017).

#### 5.1.3 Roasting

Roasting is a method where some unsoaked seeds are placed in an iron pan and heated over a low flame for 10 min. Roasting can raise the level of antioxidants by 29.1%, but simultaneously decrease the levels of polyphenols and flavonoids (Ojha et al., 2020). Thermal processing considerably affects functional properties, such as bulk density, viscosity reduction, and increased absorptivity of water and oil (Sarvani et al., 2020).

#### 5.1.4 Fermentation (LAB-based)

Fermentation is considered one of the most effective processing methods for horse gram. It is done by adding soaked seeds into a grinder to make a slurry and letting it undergo a natural fermentation process for 48 h under sterile conditions. The largest reductions in antinutritional factors are achieved by fermentation (phytic acid: 69.5%; tannins: 69.3%; and oxalates: 66.7%). Furthermore, fermentation boosts phytochemical (86.9%) and flavonoid (53.8%) content and improves antioxidant properties (Ojha et al., 2020). A synergistic effect is observed with combined processing methods, achieving tannin reduction of 61.3% and phytate reduction of 54.1% (Sarvani et al., 2020).

### 5.2 Advanced technology

Many advanced processes have been used to increase the nutritional content and remove the antinutritional elements from horse gram. Thermal treatments such as microwave cooking, infrared micronization, extrusion, and pressure cooking greatly reduce tannins, phytates, oxalates, trypsin inhibitors, and oligosaccharides while improving protein digestion and nutrient absorption. Enzymatic milling enhances extraction and reduces tannin and phytic acid content of horse gram. Similarly, sprouting and autoclaving improve protein quality, amino acid composition, and digestibility, while decreasing the antinutritional content. Thus, advanced processes provide an effective way to enhance the nutritional and functional qualities of horse gram for food use (Vashishth et al., 2021; Venketeish et al., 2026). **Table 6** shows the effects of different advanced processing on nutritional and anti-nutritional factors.

**Table 6:** Effects of advanced processing on nutritional and antinutritional factors.

Processing method	Protein/nutritional changes	Total phenolics/flavonoids	Tannins reduction	Phytate reduction	Other antinutritional changes	Antioxidant activity	References
Pressure cooking (PC)	Improved protein digestibility and amino acid availability	Flavonoids reduced by 43.9% (GPM-6) and 42.4% (PAIYUR-2)	Reduced by 66.4% (GPM-6) and 65.3% (PAIYUR-2)	Reduced by 45.5% (GPM-6) and 38.8% (PAIYUR-2)	Trypsin inhibitor reduced by 81.7%–79.3%; oxalate reduced by 78.6%–72.6%	CUPRAC activity increased by 133%–118% over raw grain	(Vashishth et al., 2021)
Pressure cooking + flaking (PCF)	Enhanced starch digestibility and nutrient bioavailability	Flavonoids reduced by 47.0% (GPM-6) and 47.5% (PAIYUR-2)	Reduced by 64.5% (GPM-6) and 64.4% (PAIYUR-2)	Reduced by 41.1% (GPM-6) and 36.2% (PAIYUR-2)	Trypsin inhibitor reduced by 81.2%–79.0%; oxalate reduced by 78.2%–72.3%	CUPRAC activity increased by 100%–93% over raw grain	
Microwave processing (MWC)	Improved protein digestibility with minimal nutrient loss	Flavonoids reduced by 39.4% (GPM-6) and 39.9% (PAIYUR-2)	Reduced by 60.4% (GPM-6) and 59.3% (PAIYUR-2)	Reduced by 53.9% (GPM-6) and 40.2% (PAIYUR-2)	Trypsin inhibitor reduced by 79.3%–78.7%; oxalate reduced by 60.3%–48.4%	CUPRAC activity increased by 81%–69% over raw grain	
Infrared micronization (IRC)	Retained nutritional quality and improved starch digestibility	Flavonoids reduced by 43.6% (GPM-6) and 41.8% (PAIYUR-2)	Reduced by 51.2% (GPM-6) and 58.5% (PAIYUR-2)	Reduced by 39.1% (GPM-6) and 32.6% (PAIYUR-2)	Trypsin inhibitor reduced by 78.3%–76.6%; oxalate reduced by 50.0%–32.7%	Highest antioxidant activity among processed samples; CUPRAC increased by 225%–181%	
Extrusion Cooking	Increased biological value and protein digestibility; slight reductions in some essential amino acids	Flavonoids reduced by 61.3% (GPM-6) and 58.2% (PAIYUR-2)	Reduced by 67.5% (GPM-6) and 65.6% (PAIYUR-2)	Reduced by 59.8% (GPM-6) and 53.3% (PAIYUR-2)	Trypsin inhibitor reduced by 81.9%–82.3%; oxalate reduced by 81.8%–86.6%; raffinose reduced by 55%–61%; stachyose reduced by 49%; verbascose reduced by 61%–62%	Antioxidant activity decreased substantially due to phenolic degradation	
Enzymatic milling (cellulase + pectinase + xylanase, 80 mg/100 g)	Protein retained with improved digestibility and enhanced nutritional quality	–	Reduced to 204.02 mg/100 g	Reduced to 204.02 mg/100 g	Significant reduction in antinutritional factors due to enzymatic degradation of hull–cotyledon matrix	Highest dhal yield (74.32%); dehulling efficiency (82.28%); cooking time reduced by 38.9% (open pan) and 85.1% (pressure cooking)	(Chandola et al., 2025)
Autoclaving (121 °C, 15 psi, 20 min)	Protein decreased to 19.16%; total amino acids decreased to 45.94 g/100 g	–	Reduced by 53.1%	Reduced by 36.1%	Trypsin inhibitor reduced by 21.1%; oxalate reduced by 74.6%	Improved digestibility, structural softening, reduced hardness, and improved functional properties	(Venketeish et al., 2026)

## 6 Physiological and biological roles

Horse gram (*Macrotyloma uniflorum*) is now considered an important functional food because of its vast source and wide scope of health-promoting bioactive and nutraceutical properties. A variety of physiological and therapeutic effects are attributed to phenolics, flavonoids, saponins, dietary fiber, peptides, and lectins acting synergistically (Arya and Divya, 2025; Oli et al., 2024). It has significant antidiabetic properties mainly due to the natural inhibition of essential carbohydrate-digesting enzymes such as α-amylase and α-glucosidase, thereby reducing the sugar hydrolysis and the postprandial rise of sugar in the blood. Concurrently, the high dietary fiber content, coupled with bioactive molecules, provides hypolipidemic properties through which the serum cholesterol levels are reduced and lipid metabolism is regulated, while enhancing bile acid excretion (Sudha and Mary Saral, 2023).

**Figure 4:**
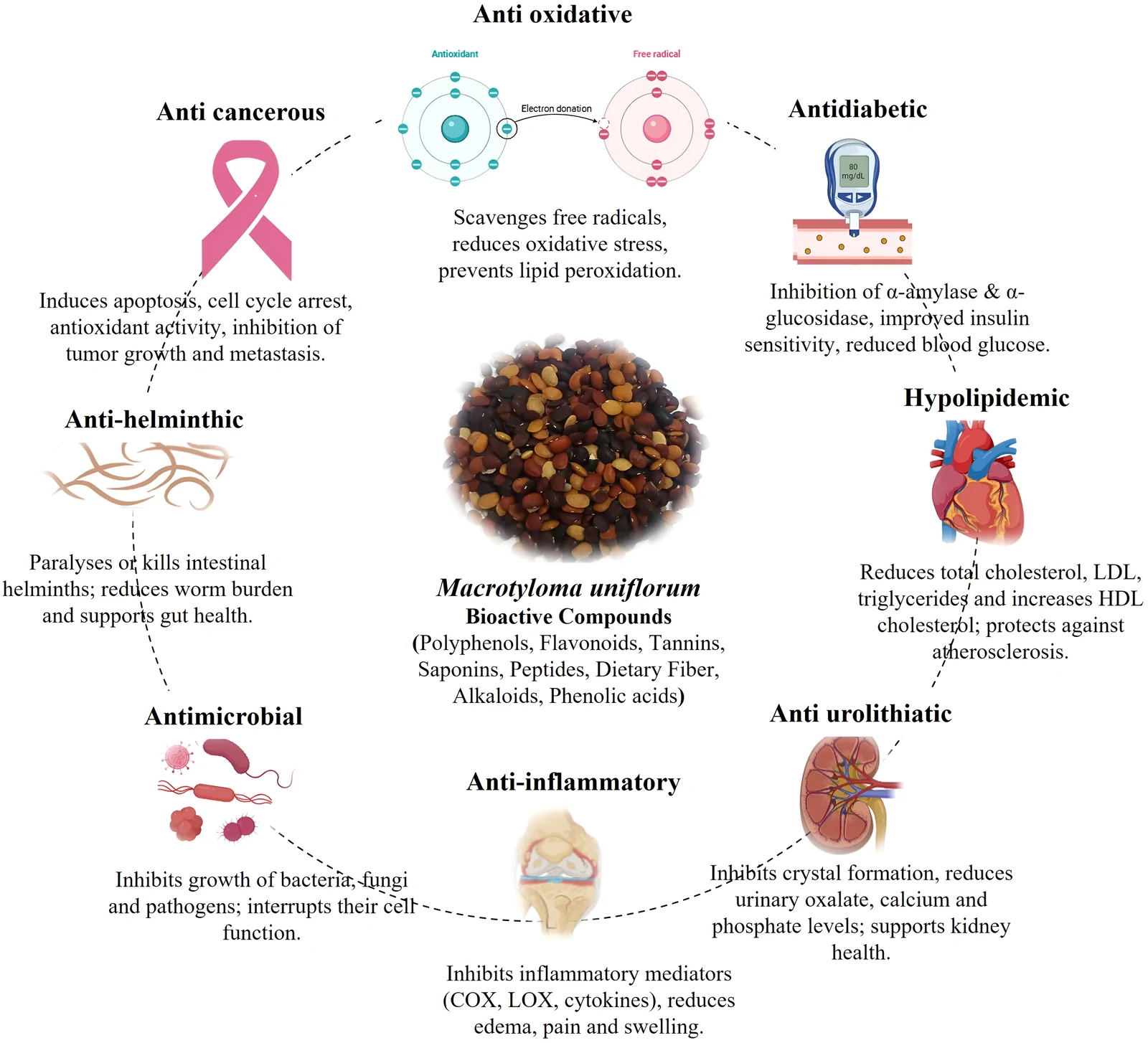
Overview of the pharmacological and health-promoting effects of horse gram seeds bioactive compounds.

Moreover, it possesses strong hepatoprotective effects, as flavonoids and other active substances are potent antioxidants that reduce oxidative stress, block lipid peroxidation, and activate hepatocyte regeneration and repair. Furthermore, due to its anti-inflammatory effects through the suppression of pro-inflammatory mediators, along with phenolic compounds and peptides, followed by its antimicrobial activity, it enhances its protective role against chronic inflammatory conditions and microbial infections (Arya and Divya, 2025; Rajagopal et al., 2017). Its reported anti-urolithiatic, immunomodulatory, digestive-enhancing, and weight-management properties add to its multifaceted nature and biological relevance. **Figure 4** shows an overview of the pharmacological and health-promoting effects of horse gram bioactive compounds.

The multinutrient properties of horse gram seed point to its potential as a traditional dietary legume and as a promising functional food, therapeutic formulation, and health-promoting nutraceutical plant resource (Arya and Divya, 2025; Malik et al., 2025; Sudha and Mary Saral, 2023). **Table 7** summarizes the pharmacological activities with experimental evidence.

**Table 7:** Summary of pharmacological activities with experimental evidence.

Activity	Experimental model	Major compound(s) involved	Key findings	References
Antidiabetic	*In vitro* α-amylase/α-glucosidase assays; streptozotocin (STZ)-induced diabetic rats (extracts, nanoparticles)	Phenolics, flavonoids, peptides, non-digestible carbohydrates	Seed extracts and HG-AgNPs inhibited carbohydrate-digesting enzymes *in vitro*, lowered blood glucose levels, and improved insulin/glucose parameters in STZ-rats, indicating hypoglycemic activity.	(Sudha and Mary Saral, 2023; Tan and Chong, 2024)
Hypolipidemic	Hyperlipidemic or diet-induced rodent models treated with seed extracts	Phytosterols (stigmasterol), polyphenols	Treatment reduced serum total cholesterol and triglycerides and improved HDL/LDL ratios in animal studies, consistent with hypolipidemic effects attributed to phytosterols and polyphenols.	(Arya and Divya, 2025; R et al., 2021)
Antioxidant activity	*In vitro* DPPH, ABTS, FRAP assays; measurement of SOD, catalase, GSH, and MDA in animal tissues	Phenolic acids, flavonoids, vitamin E, inositol derivatives	Crude extracts showed strong radical-scavenging (DPPH/ABTS) activity *in vitro* and increased SOD, catalase, and GSH while lowering MDA in treated animals, supporting antioxidant capacity.	(Arya and Divya, 2025; Sudha and Mary Saral, 2023)
Anti-urolithiatic activity	*In vitro* nucleation/aggregation assays; ethylene glycol or stone models in rats (extracts)	Tannins, saponins, polyphenols	Seed extracts inhibited calcium oxalate crystallization and reduced stone formation and urinary oxalate levels in experimental models, supporting traditional use against kidney stones.	(Sharma et al., 2019b; Sudha and Mary Saral, 2023)
Anti-inflammatory activity	*In vitro* (NO, cytokine assays in macrophages) and carrageenan/chemical-induced paw edema in rodents	Flavonoids, phenolic compounds	Extracts decreased NO and pro-inflammatory cytokine production *in vitro* and reduced paw edema and inflammatory markers *in vivo*, indicating anti-inflammatory action.	(Arya and Divya, 2025; Oli et al., 2024)
Antimicrobial	*In vitro* disc diffusion and MIC tests against bacteria and fungi (seed extracts)	Tannins, phenolics, lectins	Crude extracts exhibited inhibitory activity against selected Gram-positive and Gram-negative bacteria and some fungi, with zones of inhibition and measurable MICs reported.	
Anti-helminthic activity	*In vitro* or *in vivo* anthelmintic assays against model helminths (extract preparations)	Tannins, lectins, protease inhibitors	Seed preparations caused paralysis and death of model worms *in vitro* and reduced worm burden in animal models, supporting traditional anthelmintic claims.	
Anticancer	*In vitro* cytotoxicity assays (cancer cell lines), limited *in vivo* studies	Phenolics, flavonoids, protease inhibitors	Some fractions/extracts produced dose-dependent cytotoxicity against selected cancer cell lines and modulated apoptosis markers *in vitro*; mechanistic and *in vivo* anticancer evidence remains limited.	

### 6.1 Antioxidant activity

The phenolic acids, flavonoids, and tannins of horse gram contribute to its high antioxidant activity. These bioactive molecules have potent free-radical scavenging properties, which allow them to act as scavengers for reactive oxygen species and alleviate oxidative stress. As a result, they are associated with a decreased risk of cardiac diseases, age-related disorders, and oxidative damage to cells. The antioxidant profile of horse gram also highlights its potential as a functional ingredient to combat oxidative stress-mediated pathologies (Pal et al., 2016; Rajagopal et al., 2017; Sreerama et al., 2010).

Antioxidant activities of horse gram work by scavenging free radicals and strengthening endogenous defense mechanisms. It has been shown that horse gram increases the activities of superoxide dismutase (SOD), catalase (CAT), glutathione peroxidase (GPx), and glutathione reductase (GR), along with increasing the level of glutathione (GSH) and reducing malondialdehyde (MDA) levels. This shows that oxidative stress and lipid peroxidation can be lowered by horse gram (Rajagopal et al., 2017; Shivaji Kharat et al., 2015).

### 6.2 Anti-urolithiatic activity

Traditionally, horse gram has been used to treat kidney stones and has exhibited anti-urolithiatic activity when given in water extracts, where it has been shown to increase urine volume, reduce the concentrations of calcium and oxalates, and prevent the formation of calcium oxalate stones induced by EG in rats. It is also associated with increased levels of urolithiatic inhibitors (citrate and magnesium). It has been confirmed that extracts of *M. uniflorum* seeds inhibit the aggregation, proliferation, and crystallization of calcium oxalate, whereas they increase crystal solubilization in *in vitro* studies (Sah et al., 2024; Sharma et al., 2019a).

Anti-urolithiatic activity is believed to result from the ability of the seed to regulate urinary chemistry by increasing the urinary flow of oxalate and calcium, which are the chief components of renal stones. This is believed to help decrease supersaturation and aggregate crystal formation, which ultimately decreases the risk of stone formation (Sudha and Mary Saral, 2023). Evidence from Prasad and Singh (2015) helps support horse gram’s ethnobotanical association with renal health management.

### 6.3 Anti-inflammatory activity

Horse gram extract can help in wound management and has anti-inflammatory properties. Treatment of experimental wounds in rats showed that it accelerated wound healing and exerted anti-inflammatory effects, as reflected by the inhibition of cyclooxygenase-2 and inducible nitric oxide synthase expression. It has remarkable anti-inflammatory activity due to its flavonoids, saponins, and alkaloids (Sah et al., 2024). These bioactive compounds are effective because of their ability to regulate inflammatory signaling pathways, including the inhibition of pro-inflammatory cytokines, such as tumor necrosis factor-alpha (TNF-α) and interleukin-6 (IL-6). This molecular activity supports its potential use in traditional medicine for treating inflammation-related diseases, including arthritis and chronic inflammatory diseases (S et al., 2015). The anti-inflammatory activity also highlights the therapeutic importance of horse gram-derived compounds (Rajagopal et al., 2017).

### 6.4 Antidiabetic/hypoglycemic activity

Horse gram has been found to have potential for controlling postprandial hyperglycemia in type 2 diabetes. It contains fiber molecules, which lower the amount of glucose released into the bloodstream. The antidiabetic activity of horse gram is primarily achieved by enzyme inhibitory activity. Polyphenol-rich extracts show strong inhibition of α-amylase and α-glucosidase, which inhibits the digestion and absorption of carbohydrates. This glucose dynamic modulation leads to better glycemic control and a lower increase in blood glucose levels. Therefore, horse gram may serve as a natural remedy in the management of diabetes mellitus and glycemic control (Tiwari et al., 2013).

It has antidiabetic activity mainly due to the inhibition of α-amylase and carbohydrate-digesting enzymes, leading to delayed glucose absorption (absorption phase) and suppression of postprandial hyperglycemia. Horse gram bioactive compounds also inhibit protein tyrosine phosphatase 1B (PTP1B) and expand insulin signaling pathways, which can lead to improved insulin sensitivity and glycemic control, resulting in reduced oxidative stress processes and lower inflammatory responses (Gupta et al., 2011; Lalitha et al., 2020).

### 6.5 Hypolipidemic activity

Horse gram extracts have been observed to lower high levels of low-density lipoprotein cholesterol (LDL-C) and triglycerides, which are linked with hyperlipidemia and cardiovascular diseases (Shobana et al., 2012). In rats, the effect of *M. uniflorum* extracts resulted in a significant decrease in triacylglycerol, bad cholesterol (LDL and VLDL) and total cholesterol, along with an increase in good cholesterol (HDL). This property is related to the presence of phenolic components in the extract, which increase the breakdown of cholesterol and decrease its absorption from the intestines (Vadivelu et al., 2019).

The hypolipidemic effects of horse gram are attenuated via regulation through the phosphorylation of crucial metabolic regulators such as AMP-activated protein kinase (AMPK-α), peroxisome proliferator-activated receptors (PPAR-α and PPAR-gamma), and adipokine signaling pathways. These effects are accompanied by improved lipid metabolism, anti-inflammatory effects through the suppression of NF-kB and IL-6 synthesis, and amelioration (via modulation) in serum lipid profiles, with reduced levels of total cholesterol, triglycerides, and VLDL-cholesterol and elevated levels of HDL-cholesterol (Malarvizhi et al., 2021).

### 6.6 Anticancer activity

Recently, it has been discovered that certain molecules, such as lectins, polyphenols, and peptides, in horse gram possess strong anticancer activity. Such compounds are cytotoxic and antiproliferative against various cancer cells, through cell cycle modulation, induction of apoptosis, and inhibition of cell proliferation pathways involved in tumor growth. Preliminary investigations suggest promising potential for the therapeutic use of horse gram in oncology research (Oli et al., 2024).

The cytoprotective mechanisms of horse gram with antitumor activity are through the management of apoptosis and cell-cycle progression. The cytotoxic effects of phenolic extracts of Macrotyloma uniflorum against various cancer cell lines have been shown to result from the induction of dose-dependent apoptosis (caspase-3–mediated apoptotic cell death) and G0/G1 cell-cycle arrest. These effects are associated with histone deacetylase (HDAC) modulation, suppression of cellular proliferation, and activation of programmed cell death pathways. Furthermore, horse gram phytochemicals have been reported to enhance HDAC inhibitory activity and autophagy, suggesting their potential role in sensitizing cancer cells to therapeutic interventions (Rizwan et al., 2026).

### 6.7 Antimicrobial and anti-helminthic activity

Phenolic compounds and antimicrobial peptides are mainly considered for the antibacterial effects of horse gram. They are active against various pathogenic microorganisms through their ability to disrupt microbial cell walls, interfere with metabolic pathways, and inhibit the growth of microorganisms. This study shows the importance of horse gram as a natural antimicrobial agent and highlights its potential application in food preservation and therapeutic formulations (Parvathiraj et al., 2015). It shows strong antimicrobial activity against a range of Gram-positive and Gram-negative bacteria, such as Bacillus subtilis, Staphylococcus aureus, Escherichia coli, Pseudomonas aeruginosa, Serratia sp., Salmonella sp., and Klebsiella sp (Rao et al., 2019).

The seed also has anti-helminthic properties; its consumption may help eliminate worms, and alcoholic extracts were found to be equally effective as other known drugs such as albendazole, which is used to treat patients with worms (Khan et al., 2011).

## 7 Techno-functional properties

The techno-functional properties of horse gram flour influence its application in various food systems. The water absorption capacity of flour is very high, making it well suited for bakery products and dough formulation, enhancing dough viscosity and texture. It forms cohesive networks due to its gelation property, which is relevant in structuring agents for processed foods (Sarvani et al., 2020). Recent studies have demonstrated that germination significantly enhances the gelation capacity, rheological behavior, and structural functionality of horse gram flour, indicating its potential application in texture-modified and protein-enriched food systems (Medhe et al., 2025; Narwal and Yadav, 2022). According to rheological studies (Ghumman et al., 2016), horse gram starch gel formation is superior to that of lentil starch gels, as evidenced by higher storage (G’) and loss (G”) moduli. Nonetheless, lentil globulins have demonstrated superior protein gel-forming capacity compared to horse gram globulins upon cooling. In addition, horse gram starch gels have been found to retrograde more often, which implies that a strong gel network is formed upon storage. Although soy and pea proteins have proven their efficiency in terms of protein gelation and are commonly used in food formulations for this purpose, there has been no research on horse gram protein gelation properties. The value of water absorption capacity obtained was 142.14 g/100 g, which shows that it has a great affinity for water due to the constituents of horse gram, including dietary fiber and protein content, which improve the viscosity and functional properties in a water medium. Its foaming and emulsifying properties also allow its application in beverages, aerated products, and emulsion-based foods. Interestingly, enhancement in these functional attributes after processing treatments, such as germination and fermentation, has been observed in horse gram, as the foam capacity was 38.16% and foam stability was 35.12%, indicating excellent foam-forming capacity, which can be attributed to the ease of mobility of proteins in solution. These properties make horse gram suitable for aerated food products. Furthermore, the emulsion capacity (52.15%) and emulsion stability (50.32%) indicate the capability of horse gram proteins as excellent stabilizers at the oil-water interface for formulating emulsified products such as dressings, sauces, and plant-based products (Sarvani et al., 2020).

Horse gram flour has been found to have functional properties, indicating its suitability for various applications in food. The granule expansion value of horse gram was found to be 0.46, with a suitable hydration capacity of swell volume of 1.43 mL, which shows moderate water absorption capacity, along with granule expansion when water is added. The oil absorption capacity (80.76%) is comparatively high in flour, and it is desirable for formulated food products to have high OAC, which is facilitated by the presence of non-polar amino acids that bind to lipids and help improve the mouthfeel and flavor of formulated products (Marimuthu and Krishnamoorthi, 2013).

The water solubility index (7.56%) also indicates the presence of macromolecules that are soluble in water, supporting dispersion in foods, such as soluble fiber and denatured proteins.

Compared to other common legume proteins, horse gram has better techno-functionality for use in batter and semi-solid food systems due to its water absorption, swelling, emulsification and gelation capacities. However, limited research has been conducted on gelling in horse gram compared to soy or pea proteins. Gelation of horse gram has been documented in some literature showing gelation at low concentrations; however, information on gel strength, rheology, and gel microstructure is limited. Soy protein is generally regarded as the standard for gels with strong heat-induced gelation, while gels of pea protein are normally weaker than those of soy protein. Due to its favorable techno-functionality, horse gram may be classified as a functional legume protein, but more studies are required to determine the gelling capacity of horse gram in comparison to soy and pea proteins in applications like meat alternatives, desserts, and other structured protein-based foods (Banerjee et al., 2022; Haripriya et al., 2017; Medhe et al., 2025; Shrestha et al., 2023). These results show horse gram could be a functional food ingredient for products that rely on gel formation. Further studies may be required on gel strength, water-retention capacity, and the microstructure of horse gram gel to determine if it could be used for more products in different industries.

## 8 Applications in food development

Functional food development of horse gram (*Macrotyloma uniflorum*), a nutritious leguminous crop, has attracted considerable attention in recent years based on its enhanced nutritional value and bioactive properties. Innovative product developments have shown the potential use of horse gram in improving the functional properties, sensory characteristics, and nutritional value of foods.

An application of interest is the use of ready-to-eat snack foods prepared with 30% malted horse gram flour mixed with corn, wheat, and rice flour. The use of chemical (tanning), biological (germination), and physical (controlled drying) methods was effective in decreasing the levels of antinutritional factors, including tannins by 39.1%, along with improving nutritional content of protein (17.8%), calcium (0.22), and iron (0.08%). The sensory attributes of these processed products are also remarkable and compatible with safety requirements, with an absence of any microbial growth, indicating the possibility of horse gram utilization in convenient snack foods (Nazim et al., 2024; Rana et al., 2023). The impressive bioactive compound, protein, and fiber content of horse gram, along with its promising nutritional profile, provide good potential for its use as a functional ingredient in sustainable food products, including plant-based meat alternatives. Devi et al. (2024) prepared a horse gram-based plant-based meat-like product with soy flour and wheat flour and optimized the product to enhance its nutritional assimilation, texture, and sensory acceptability. A new niche is the formation of a functional milk beverage where 5%–15% of horse gram extract was added to cow milk, dahi, banana, and black carrot juice, and smoothies were prepared. Research revealed that formulations containing 10% horse gram produced the most optimum nutritional and functional qualities, with 3.38% protein, 22.17% carbohydrate, and high antioxidant activity (54%). Furthermore, the smoothie showed desirable viscosity and low syneresis, with a refrigerated shelf life of 9 days, which will encourage the development of the smoothie as a functional dairy-based product (Panda et al., 2023). It was found that 2%–6% fortification of horse gram improved nutrition content in Greek yogurt without compromising its sensory quality, and 15%–20% fortification of horse gram in chapati, biscuit, and burfi increased nutritional value (Protein ↑0.53%–6.07%; Ca ↑24%–58.66%; Fe ↑0.38%–2.17%) along with acceptable shelf life (Venugopal and H, 2025). Horse gram has also been incorporated into the preparation of fortified protein cookies, in which the wheat flour was replaced to some extent by 25%–50% germinated horse gram protein extract. An increase in protein content with no deterioration in sensory acceptability and textural quality gives rise to improved nutritional value properties in these cookies. Such improvements indicate the potential use of horse gram as a protein-enhancing ingredient in bakery products (Narwal and Yadav, 2025). In addition to food applications, the properties of horse gram proteins have also been investigated in biodegradable packages. Horse gram protein films have been made transparent by citric acid crosslinking and used for potential application in food packaging and biomedical applications. The citric acid content obtained was as high as 15%, which drastically lowered the water sorption (25%), increased tensile strength (2.24 to 5.3 MPa), and increased the antioxidant activity (96%) and antimicrobial properties. Such films showed good thermal stability and cytocompatibility, which hold great promise for applications in sustainable packaging and medical substrates (Sakkara et al., 2020).

Besides research-based developments, several commercial food products based on horse gram have also been introduced to the market, ranging from horse gram flour mixes, porridge mixes, protein soups, instant noodles, idly powders, and formulations for the intake of dietary supplements. The nutritional advantages of horse gram and its acceptance in the functional or convenience food markets are driving greater interest in these products (Malik et al., 2025). **Table 8** shows functional food applications of horse gram.

**Table 8:** Functional food applications of horse gram.

Product type	Horse gram form used	Functional benefit	Industrial potential	References
Bakery products	Cold plasma-treated flour	Antioxidant and anti-inflammatory properties; reduced antinutritional factors (tannins and phytic acid); enhanced digestibility (75%)	Viable ingredient for baking/confectionery; improved dough characteristics and emulsifying properties	(Patra et al., 2024)
Beverages	Plant-based milk; milk-based smoothie with extract (5%–15%)	Antioxidant activity (31.11% DPPH inhibition), antidiabetic and anti-urolithiatic properties; high protein (22%–25%), dietary fiber, and polyphenols	Sustainable dairy alternative; 9-day shelf life under refrigeration; addresses growing demand in the global plant-based dairy sector	(Panda et al., 2023; Reddy and Uthira, 2026)
Snack products	Protein hydrolysates; fortified snacks	Enhanced digestibility and bioavailability; antioxidant and antidiabetic properties; high lysine and micronutrients	High-protein snacks, therapeutic diets, geriatric nutrition applications	(Panda et al., 2023; Rana et al., 2023)

## 9 Future prospects

Horse gram (*Macrotyloma uniflorum*) could serve as a sustainable functional food crop, but more research is needed to fully realize its potential. Future investigations should include human clinical trials in order to test its reported pharmacological effects, such as antidiabetic, antiobesity, and anticancer properties. Furthermore, extensive *in vivo* metabolic and bioavailable studies of its bioactive compounds are necessary to elucidate its efficacy *in vivo*. Other process optimization and standardization methods, such as germination, fermentation, and roasting, are also needed to ensure that stocks are maximized while antinutritional factors are reduced. The use of new technologies, such as omics technologies (metabolomics and proteomics), can aid in the identification of novel bioactive compounds and improve the understanding of their mechanisms of action through advances in molecular biology. Moreover, extensive opportunities exist for novel functional food products, protein-based food products, and nutraceuticals using horse gram as an ingredient. It is important for future researchers to study how nutrigenomics and gene–diet interactions help us understand how horse gram’s beneficial effects on health occur at a molecular level. This will allow the development of personalized diets and precision functional foods designed to meet the specific health needs of individuals. Breeding efforts aimed at improving agronomically important characteristics, such as yield, nutrient content, and stress tolerance, will further pave the way for its cultivation in greater numbers. The fact that horse gram can grow in areas where there are few resources (i.e., it can survive and produce well during times when other crops would fail because of water scarcity) means that horse gram can be grown in drought-affected regions of sub-Saharan Africa, the Middle East, Australia, and Latin America, thereby making positive contributions to our ability to provide food and nutrition. Furthermore, it has potential applications in biodegradable packaging and pharmaceutical formulations, introducing new industrial opportunities. In conclusion, the incorporation of horse gram into modern food systems holds the promise of enhancing nutritional security, sustainable agriculture, and healthy food production.

**Figure 5:**
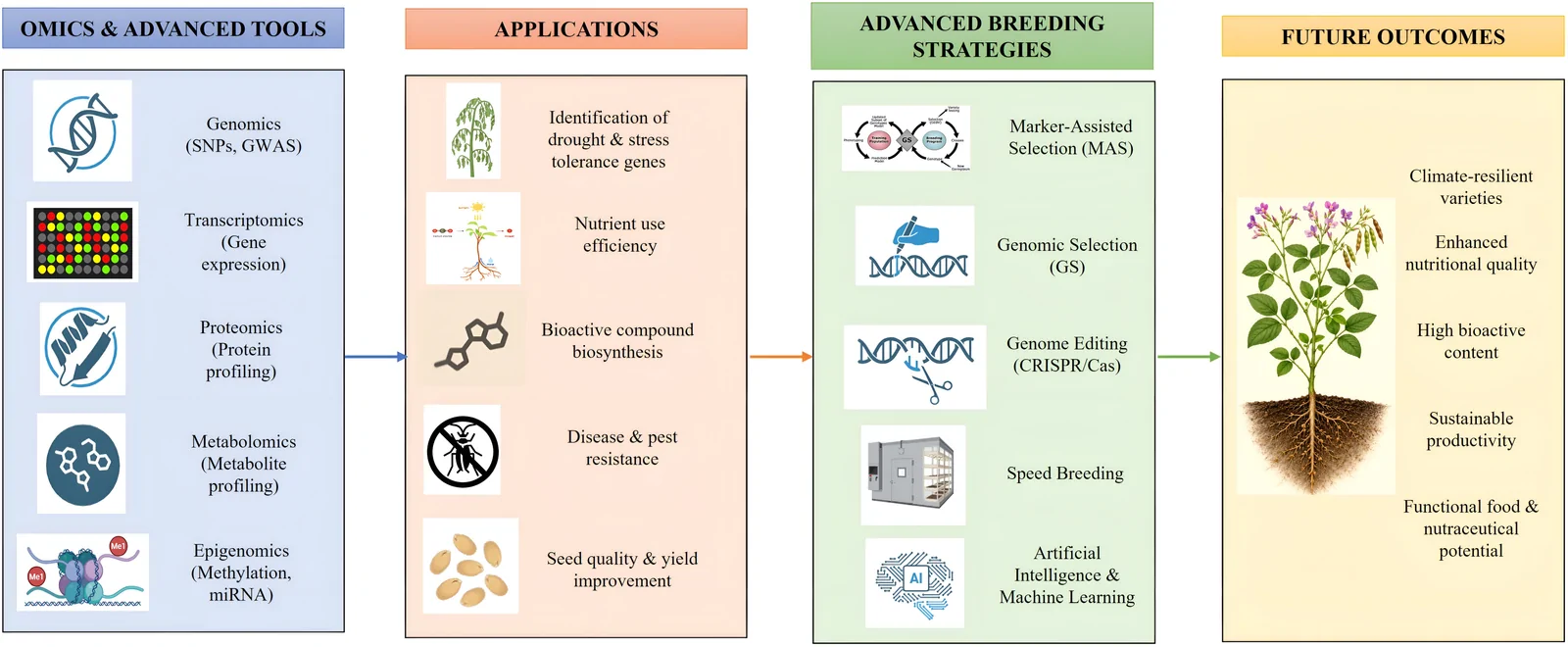
OMICS-assisted breeding and future research road map for horse gram seeds.

**Figure 5** shows, and **Table 9** explains, the omics and advanced research opportunities for horse gram.

**Table 9:** Omics and advanced research opportunities in horse gram.

Omics/research area	Current status in horse gram	Advanced research opportunities	Potential application	Key references
Whole genome sequencing	Draft genome and genome annotation available	Chromosome-level assembly, pan-genomics	Discovery of elite alleles and stress genes	(Mahesh et al., 2021)
Transcriptomics	RNA-seq and transcriptome profiling reported	Single-cell transcriptomics, long-read transcriptomics	Identification of stress-responsive pathways	(Mahesh et al., 2021)
Functional genomics	Candidate genes identified for disease resistance and drought adaptation	CRISPR/Cas gene editing, functional validation	Development of resistant cultivars	(Mahesh et al., 2021)
Molecular marker development	SSR, EST-SSR, ISSR, and SNP markers developed	High-density SNP arrays and KASP markers	Marker-assisted breeding	(Chahota et al., 2017; Pratihari and Jegadeesan, 2026)
Genome-wide association studies (GWAS)	SNP-based GWAS conducted for agronomic traits	Multi-environment GWAS and genomic prediction	Identification of trait-linked loci	(Sharma and Chahota, 2025)
QTL mapping	Initial QTL studies available	Fine-mapping and candidate gene identification	Precision breeding	(Chahota et al., 2017; Sharma and Chahota, 2025)
Proteomics	Limited proteomic exploration	Stress-responsive proteomics and phosphoproteomics	Protein biomarkers for stress tolerance	(Mahesh et al., 2021)
Metabolomics	Extensive phytochemical and antioxidant profiling reported	LC-MS/MS metabolomics and pathway engineering	Functional food development	(Bhartiya et al., 2015; Sreerama et al., 2010)
Epigenomics	Very limited information available	DNA methylation and histone modification studies	Understanding stress memory	(Mondal and Kharwal, 2024)
Phenomics	Conventional phenotyping widely used	AI-based high-throughput phenotyping	Rapid screening of elite genotypes	(Pratihari and Jegadeesan, 2026)
Bioinformatics and systems biology	Multi-omics integration initiated	Network biology and predictive modeling	Gene-to-trait association studies	(Mondal and Kharwal, 2024)
Speed breeding and genomic selection	Conceptual stage in horse gram breeding	Accelerated generation advancement	Faster varietal development	(Mondal and Kharwal, 2024)
Microbiome research	Limited research available	Rhizosphere microbiome engineering	Improved nutrient uptake and stress tolerance	(Bhardwaj et al., 2013)
Climate-resilient breeding	Horse gram recognized as drought-tolerant and climate-resilient	Multi-stress omics-assisted breeding	Adaptation to climate change	(Mahesh et al., 2021; Mondal and Kharwal, 2024)
Nutrigenomics and functional foods	Nutraceutical and medicinal properties extensively reported	Biofortification and nutrigenomic studies	Development of therapeutic foods	(Bhartiya et al., 2015; Kaundal et al., 2019)

## 10 Conclusion

Horse gram (*Macrotyloma uniflorum*) is a valuable legume that can help achieve global food and nutrition security and climate change-resilient agricultural systems. It contains approximately 25%–30% protein and is rich in dietary fiber and other nutrients. It contains different bioactive components with diverse functional and nutraceutical properties. For example, these components exhibit antioxidant, antidiabetic, cardioprotective, and anti-inflammatory activities. The grains can undergo different processing techniques, such as germination, fermentation, roasting, and advanced thermal techniques, to improve their nutrient bioavailability and reduce their antinutritional factors, thereby making them more suitable for food use. With the growing interest in plant-based ingredients, horse gram can be utilized in the preparation of functional foods, nutraceuticals, and other value-added products. Although bioavailability and other concerns regarding mechanistic studies, processing, and consumer education pose challenges, future research should focus on human clinical studies, evaluating horse gram processing at an industrial scale, measuring economic viability, and clarifying regulatory pathways to support the development of functional foods and nutraceuticals based on horse gram. Horse gram has the potential to achieve greater acceptance and commercialization.
